# Blood pressure measurement: Should technique define targets?

**DOI:** 10.1111/jch.14324

**Published:** 2021-07-16

**Authors:** Swapnil Hiremath, Tim Ramsay, Marcel Ruzicka

**Affiliations:** ^1^ Department of Medicine University of Ottawa Ottawa Ontario Canada; ^2^ Clinical Epidemiology Program Ottawa Hospital Research Institute Ottawa Ontario Canada; ^3^ Kidney Research Center University of Ottawa Ontario Canada

**Keywords:** ambulatory blood pressure/home blood pressure monitor, clinical management of high blood pressure, clinical trials, epidemiology, hypertension—general

## Abstract

Accurate assessment of blood pressure (BP) is the cornerstone of hypertension management. The objectives of this study were to quantify the effect of medical personnel presence during BP measurement by automated oscillometric BP (AOBP) and to compare resting office BP by AOBP to daytime average BP by 24‐h ambulatory BP monitoring (ABPM). This study is a prospective randomized cross‐over trial, conducted in a referral population. Patients underwent measurements of casual and resting office BP by AOBP. Resting BP was measured as either unattended (patient alone in the room during resting and measurements) or as partially attended (nurse present in the room during measurements) immediately prior to and after 24‐h ABPM. The primary outcome was the effect of unattended 5‐min rest preceding AOBP assessment as the difference between casual and resting BP measured by the Omron HEM 907XL. Ninety patients consented and 78 completed the study. The mean difference between the casual and Omron unattended systolic BP was 7.0 mm Hg (95% confidence interval [CI] 4.5, 9.5). There was no significant difference between partially attended and unattended resting office systolic BP. Resting office BP (attended and partially attended) underestimated daytime systolic BP load from 24‐h ABPM. The presence or absence of medical personnel does not impact casual office BP which is higher than resting office AOBP. The requirement for unattended rest may be dropped if logistically challenging. Casual and resting office BP readings by AOBP do not capture the complexity of information provided by the 24‐h ABPM.

## 1 INTRODUCTION

Accurate blood pressure (BP) measurement is a cornerstone for the diagnosis and management of hypertension.^1^ Technological progress over the last two decades has advanced the accuracy of BP assessment. Specifically, automated oscillometric devices eliminate most human errors in the art of measurement of BP and the interpretation of auscultation information, and provide the average of multiple BP readings and the time over which these readings are taken.^2^ Full automation of these devices now have also allowed for a standardized period of rest, including unattended rest, preceding the BP measurements.^3^

Despite this, casual office BP by automated oscillometric devices in most medical visits is still quite common. Casual office BP measurement may overestimate the true BP load because of the white coat effect.^4^ Hence, casual office BP is not recommended, neither for the diagnosis nor for the management of hypertension. Resting office BP is recommended by all professional societies, with variation in the definition of what this entails, ranging from a clear preference for automated oscillometric BP (AOBP) by Hypertension, Canada to a clear admonishment against using unattended AOBP from the European Society of Hypertension/European Society of Cardiology as well as the International Society of Hypertension.^5^, ^6^, ^7^, ^8^ In the largest randomized controlled trial (RCT) to inform clinical practice in this area, namely the Systolic blood PRessure InterveNtion Trial (SPRINT), AOBP was used for BP measurement.^9^ While the methodology of BP assessment in this trial was standardized concerning the model and manufacturer of the automated oscillometric BP device, resting time, and number of recordings, it turned out that measurements in some centers were performed as completely unattended while in others with medical personnel present for part of the measurements.^10^ The requirement for completely unattended rest may be logistically challenging for many clinical settings, and may entail the need to purchase new devices if the existing AOBP device does not allow for this. Lastly, though 24 h ambulatory BP monitoring (ABPM) provides the most comprehensive data on BP load during the day and night, diurnal patterns of BP, and BP variability, this was not performed at the same time as the office BP measurements in SPRINT, making comparisons between reported BP readings difficult.^10^

To address these issues, we conducted a prospective crossover trial to examine the effect of complete absence and partial absence of medical personnel on resting BP. In addition to that, we compared results of resting automated office BP (in the complete and partial absence of medical personnel) to corresponding office visit casual BP and average of the daytime BP from 24‐h ABPM administered at the time of office visit.

## 2 METHODS

### 2.1 Study design

This was a prospective, randomized, crossover study to compare different BP measurement methods (see Figure S1 for study flow).

### 2.2 Population

#### 2.2.1 Inclusion criteria

All patients being followed in a tertiary care, referral hypertension clinic were eligible for enrollment.

#### 2.2.2 Exclusion criteria

Patients for whom oscillometric measurements may be difficult were excluded, namely with inability to do oscillometric measurements (eg, arrhythmias, pain, device reporting error) or lack of consent from the patient.

### 2.3 Measurements

Initial casual BP was measured by a trained hypertension registered nurse, using a proper BP measurement technique, with an AOBP device. Subsequent AOBP measurement was done using the same AOBP device: either the Omron HEM 907XL (Omron Health care Inc, West Field Court Lake Forest, IL, USA) or the BpTRU (BpTRU Medical Devices, Coquitlam, British Columbia, Canada). For the Omron device, BP was measured as an average of three readings, taken 2 min apart, after a period of 5 min rest. This was performed either with the patient alone in the room (Omron Unattended) or with the nurse entering the room after the 5 min rest, and quietly working during the three measurements (Omron, Partially attended). For the BpTRU, it was measured as an average of five readings taken 2 min apart, with no initial rest period, with the patient being alone in the room. This was subsequently followed by a 24 h ABPM (SpaceLabs model 90207, SpaceLabs Health care, Snoqualmie, WA, USA). When the ABPM device was returned the next day, another set of casual and AOBP measurements were performed, with a different AOBP device or method (see Figure S1). Measurements for ABPM were done every 20 min during the awake period and every 30 min during sleep.^11^ We only accepted reports comprising at minimum 75% recordings as being valid. Awake and sleep periods were defined according to the patients’ diaries. The order of AOBP measurement methods was randomized. Randomization was done by generating a randomization list and allocation was concealed using sealed envelopes.

### 2.4 Outcomes

The primary outcome was the effect of unattended 5‐min rest preceding unattended systolic BP assessment as derived from the difference between casual BP and average resting unattended systolic BP (by Omron HEM 907XL).

There were three secondary outcome measures, namely the difference between average systolic BP measured with BpTRU and Omron unattended; the difference between the average systolic BP between the Omron unattended and partially attended methods, and the difference between the average unattended systolic BP (by Omron HEM 907XL) and daytime average systolic BP from 24‐h ABPM. *Post hoc* analyses included the same outcomes as described above for diastolic BP, and a pooled comparison of all AOBP methods with casual and daytime average BP from 24 h ABPM.

### 2.5 Analysis

Sample size estimation: The magnitude of the difference between casual and unattended average of three oscillometric readings after 5 min rest appears to be about 12.7 mm Hg based on existing literature.^12^ Our review of the literature identified a range of possible standard deviations from 5 to 16 mm Hg.^13^ To be conservative, we assumed a standard deviation of 16 mm Hg. Hence, a sample size of 55 would give 90% power with a t‐test at an alpha of 0.05. We anticipated little loss to follow up given the short nature of the study (two visits 1 day apart). In addition, we planned to enroll an additional 30 patients in whom we would compare the effect of partially attended versus completely unattended BPs. We assumed that a maximum of five patients would have errors with BP measurements with oscillometric methods. Hence we planned to enroll 90 patients in this trial.

Analytic Plan: For baseline characteristics, continuous variables are reported as mean and standard deviation, and nominal variable in percentages. The BP measurements were compared using Student's paired t‐test. Bland‐Altman analysis was performed to compare the 95% limits of agreement.^14^

The trial was registered on ClinicalTrials.gov (NCT03267420). This study was approved by the Ottawa Health Sciences Research Ethics Board.

## 3 RESULTS

**FIGURE 1:**
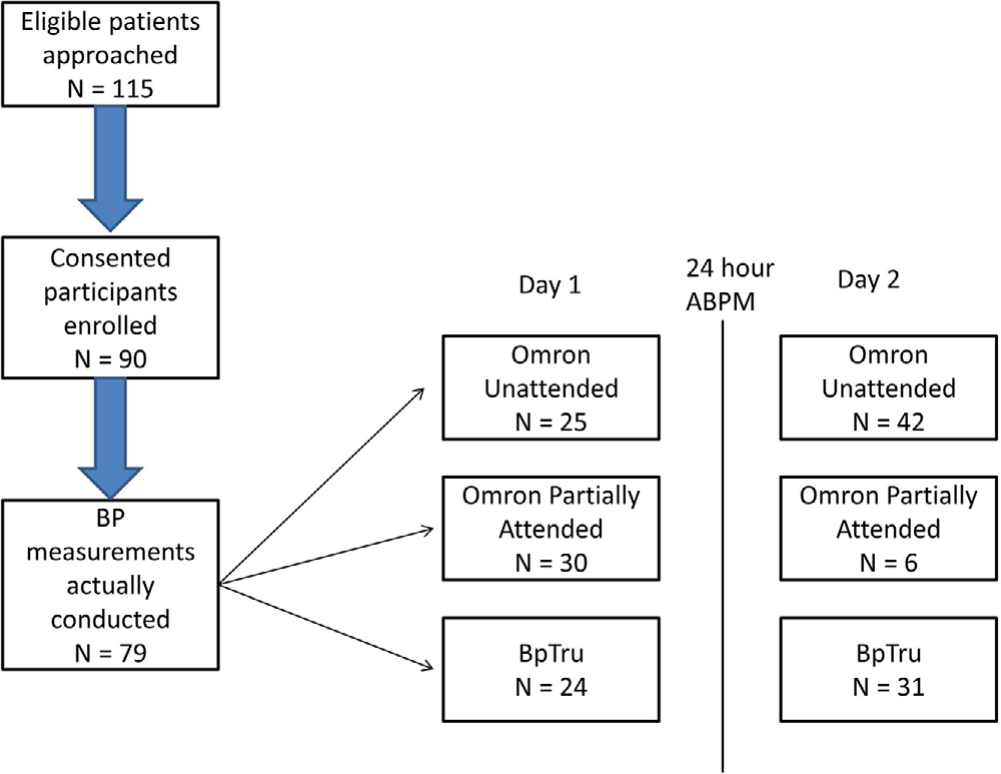
Participant flow in the study

Ninety patients consented for this study, and 78 patients completed the study as designed, over the period of study from January 2018 to January 2019 ( see details in Figure 1) . Due to protocol violations, the numbers in each group were not exactly 30 as planned, and are being reported as performed. They were middle aged with the mean age of 62 years, with 44 (56 %) men. The average body mass index was 30.4 kg/m^2^ and about a quarter had preexisting heart disease or diabetes. The other baseline characteristics are presented in Table 1.

**Table 1:** Baseline characteristics

Characteristics	Value
Age (years)	61.7 + 14.1
Men	44 (55.7%)
Arm circumference (cms)	33.2 + 4.6
BMI (kg/m^2^)	30.4 + 6.0
Ischemic heart disease	21 (26.6%)
Peripheral arterial disease	12 (15.2%)
Cerebrovascular disease	9 (11.4%)
Diabetes mellitus	20 (25.3%)
Statin use	34 (43.0%)
Current smoker	6 (7.6%)
eGFR (ml/min/1.72 m^2^)	
Number of BP lowering medications (median, IQR)	2 (1,3)

All data as mean + standard deviation or number (percentage) unless otherwise specified.

*Abbreviations*: BMI, body mass index; BP, blood pressure; IQR, interquartile range.

### 3.1 Casual BP compared to resting AOBP

The mean difference between the casual and Omron unattended systolic BP was 7.0 mm Hg (95% confidence interval [CI] 4.5, 9.5 mm Hg). The difference between the casual and Omron partially attended (mean 6.9, 95 % CI 4.5, 9.3 mm Hg) and the casual and BpTru (mean 8.3, 95 % CI 5.4, 11.3 mm Hg) was also similar. For diastolic BP, the mean difference between casual and resting AOBP varied from 2.2 mm Hg to 4.6 mm Hg (see Table 2 for more details).

**Table 2:** Differences between casual and resting blood pressure readings

	N	Method	Mean ± standard deviation	Mean difference (95% CI)	*p* value
Systolic BP					
Casual versus Omron unattended	66	Casual	137.1 ± 16.7	−7.0 (−4.5, −9.5)	<.0001
		Omron U	130.1 ± 13.7		
Casual versus Omron partially attended	32	Casual	135.0 ± 14.4	−6.9 (−4.5, −9.3)	<.0001
		Omron P	128.1 ± 12.6		
Casual versus BP Tru	52	Casual	136.5 ± 19.1	−8.3 (5.4, 11.3)	<.0001
		BP Tru	128.2 ± 15.0		
Diastolic BP					
Casual versus Omron unattended	66	Casual	71.3 ± 17.4	− 3.1 (−1.8, −4.4)	<.0001
		Omron U	68.2 ± 12.2		
Casual versus Omron partially attended	32	Casual	71.6 ± 10.1	−2.7 (−0.8, −4.7)	.008
		Omron P	68.9 ± 10.3		
Casual versus BP Tru	52	Casual	74.8 ± 12.0	−2.2 (−0.8,−3.6)	.002
		BP Tru	72.6 ± 11.5		

### 3.2 Difference between the resting AOBP methods

The mean difference between the Omron unattended and partially attended was 2.7 mm Hg (95 % CI ‐4.0, 1.4 mm Hg). Similarly, there was not a significant difference between Omron unattended and BpTRU (mean 1.4, 95% CI ‐5.6, 2.8 mm Hg) or the Omron partially attended and BpTRU (mean ‐4.5, 95 5 CI 11.5, ‐20.5 mm Hg). For diastolic BP, the mean difference was not significant between the two Omron methods at 0.5 mm Hg, but the Omron unattended reported a lower diastolic BP compared to the BpTru (mean ‐3.4, 95% CI 0.5, 6.3 mm Hg), with more details in Table 3.

**Table 3:** Differences between individual resting AOBP methods

	N	Method	Mean + SD	Mean difference (95% CI)	*p* value
Systolic BP					
Omron unattended versus Omron partially attended	22	Omron U	132.0 ± 10.7	−2.7 (−4.0, 1.4)	.18
		Omron P	129.3 ± 13.2		
Omron unattended versus BP Tru	43	Omron U	129.7 ± 14.8	1.4 (−5.6, 2.8)	.49
		BP Tru	128.3 ± 14.7		
Omron partially attended versus BP Tru	6	Omron P	129.7 ± 11.7	−4.5 (11.5, −20.5)	.50
		BP Tru	125.2 ± 19.0		
Diastolic BP					
Omron unattended versus Omron partially attended	22	Omron U	68.7 ± 10.7	0.5 (−1.9, 3.0)	.66
		Omron P	69.2 ± 9.7		
Omron unattended versus BP Tru	43	Omron U	68.6 ± 12.9	3.4 (0.5, 6.3)	.02
		BP Tru	72.1 ± 11.3		
Omron partially attended versus BP Tru	6	Omron P	67.2 ± 13.7	1.3 (−1.9, 12.5)	.12
		BPTru	72.5 ± 15.4		

### 3.3 Comparison of resting AOBPs with daytime average from 24 h ABPM

The mean systolic BP with AOBP as measured by Omron unattended was lower than the daytime ABPM average (difference – 7.8 mm Hg, 95 %CI ‐5.0, ‐10.8). Similarly, the mean systolic BP with other AOBP methods was also lower than the daytime ABPM (Omron partially unattended ‐10.8 mm Hg, 95 % CI ‐6.6, ‐15.0; BpTru ‐9.3 mm Hg, 95 % CI ‐5.9, ‐12.6). For diastolic BP, the mean difference ranged from ‐4.5 mm Hg (with BpTru) to – 9.0 mm Hg (with Omron partially attended). See more details in Table 4.

**Table 4:** Differences between resting AOBP and daytime ABPM

	N	Method	Mean + SD	Mean difference (95% CI)	*p* value
Systolic BP					
Daytime ABPM versus Omron unattended	66	ABPM	138.0 ± 10.4	−7.8 (−5.0, −10.8)	<.0001
		Omron	130.1 ± 13.7		
Daytime ABPM versus Omron partially attended	32	ABPM	138.9 ± 12.4	−10.8 (− 6.6, −15.0)	<.0001
		Omron	128.1 ± 12.6		
Daytime ABPM versus BP Tru	52	ABPM	137.4 ± 10.6	−9.3 (−5.9, −12.6)	<.0001
		BP Tru	128.2 ± 15.0		
Diastolic BP					
Daytime ABPM versus Omron unattended	66	ABPM	76.4 ± 9.9	−8.1 (− 5.8, − 10.4)	<.0001
		Omron	68.2 ± 12.2		
Daytime ABPM versus Omron partially attended	32	ABPM	77.8 ± 10.8	−9.0 (− 5.5, − 12.4)	<.0001
		Omron	68.9 ± 10.3		
Daytime ABPM versus BP Tru	52	ABPM	77.1 ± 11.3	−4.5 (− 2.7, − 6.4)	<.0001
		BP Tru	72.6 ± 11.5		

### 3.4 Comparison of casual, resting AOBP (pooled), and daytime ABPM

Casual office BP readings were significantly higher as compared to resting office BP by AOBP (see details in Table 5). The AOBP systolic and diastolic BP was significantly lower than the daytime ABPM by 10.3 mm Hg and 7.9 mm Hg, respectively.

**Table 5:** Differences between casual, pooled AOBP and daytime ABPM

	N	Method	Mean + SD	Mean difference (95% CI)	*p* value
Systolic BP					
Casual versus AOBP	79	Casual	134.0 ± 16.8	6.5 (4.3, 8.7)	<.0001
		AOBP	127.5 ± 13.5		
Casual versus ABPM	79	Casual	134.0 ± 16.8	−3.8 (− 0.7, 6.9)	.02
		ABPM	137.8 ± 11.0		
AOBP versus ABPM	79	AOBP	127.5 ± 13.5	−10.3 (− 7.9, −12.7)	<.0001
		ABPM	137.8 ± 11.0		
Diastolic BP					
Casual versus AOBP	79	Casual	71.9 ± 11.2	2.7 (1.5, 3.7)	<.0001
		AOBP	69.2 ± 11.3		
Casual versus ABPM	79	Casual	71.9 ± 11.2	−5.2 (− 3.5, −7.0)	<.0001
		ABPM	77.2 ± 10.5		
AOBP versus ABPM	79	AOBP	69.2 ± 11.3	−7.9 (− 6.0, − 9.7)	<.0001
		ABPM	77.2 ± 10.5		

### 3.5 Limits of agreement

**FIGURE 2:**
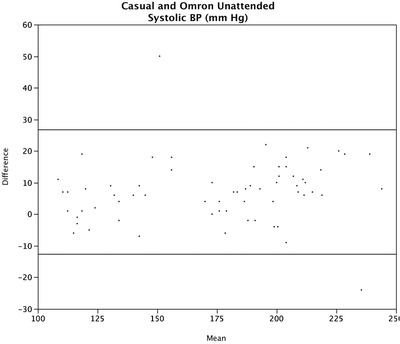
Bland Altman limits of agreement for casual compared to Omron unattended systolic blood pressure

The detailed limits of agreement using the Bland‐Altman method are presented in Table 6. These were 26.7 to ‐12.7 mm Hg for casual compared to Omron unattended systolic BP (Figure 2) and 15.0 to ‐32.8 for pooled AOBP compared to daytime ABPM (Figure S2).

**Table 6:** Bland Altman limits of agreement

Comparison	95% limits of agreement *Systolic BP (mm Hg)*	95% limits of agreement *Diastolic BP (mm Hg)*
Casual and Omron unattended	26.7, −12.7	13.2, −7.0
Casual and Omron partially attended	20.0, −6.2	13.1, −7.7
Casual and BpTru	29.2, −12.5	11.9, −7.4
Omron unattended and partially attended	20.7, −15.3	10.0, −11.1
Omron unattended and BpTru	28.2, −25.3	15.0, −21.9
Omron partially attended and BpTru	34.4, −25.4	8.1, −18.8
Daytime ABPM and Omron unattended	15.3, −31.0	9.3, −27.3
Daytime ABPM and Omron partially attended	12.1, −33.7	10.1, −26.3
Daytime ABPM and BpTru	14.6, −33.1	8.4, −17.4
Casual and pooled AOBP	28.0, −17.7	12.5, − 7.0
Casual and ABPM	31.2, −23.6	10.1, −20.5
ABPM and pooled AOBP	15.0, −32.8	9.9, −25.9

## 4 DISCUSSION

This prospective randomized crossover trial found that office BP readings are significantly higher compared to office BP readings after a 5‐min rest. As treatment thresholds and BP targets in most guidelines are based on research quality resting office BP, it is important to acknowledge that the unattended resting described in the trials may impose logistical issues for practitioners, with no clinically meaningful impact on true resting office BP. Casual and resting office BP readings by AOBP naturally could not capture the complexity of BP behavior, and overall daytime and nocturnal BP load provided by 24‐h ABPM and as such, they should not be the sole diagnostic tool for diagnosis and management of hypertension.

In the SPRINT trial, a fully automated oscillometric ambulatory BP device, the Omron HEM907 XL, was used for the assessment of office BP.^9^, ^10^ This device allows for a specified period of unattended rest prior to sequential BP measurements from which the resting office BP is averaged. Until then devices utilized worldwide for the assessment of BP may have required medical personnel to enter the room to switch the device on after the defined resting time. The difference between these two methods is obvious with regards to perceived potential effect on BP.^15^, ^16^ Firstly, fully automated oscillometric BP device allows for truly unattended resting and measurement of BP. Secondly, the resting time is also standardized and controlled by a fully automated oscillometric BP device. Furthermore, as described in the introduction, the actual procedure of obtaining resting office BP in the SPRINT differed (from completely unattended measurements to partially attended measurements) between centers.^10^ Hence it was important to assess to what extent BP readings obtained by these two methods differ (or not) from each other. Our results showed that BP readings obtained by fully unattended versus partially attended oscillometric BP technique are similar from both statistical and clinical point of view. Our data are in agreement with others.^17^, ^18^ For example, Andreadis and coworkers showed that in 146 middle aged (mean age 56±12 years) patients with a diagnosis of hypertension, the difference between attended and unattended systolic BPs obtained by Omron HEM 907 XL was 0.6 ± 6 mm Hg.^18^ In contrast, a meta‐analysis of 12 clinical studies comprising 1762 patients done by the same group indicated lower unattended compared to attended automated office systolic BPs measurement [by ‐3.66 (‐6.58 to ‐0.75) mm Hg].^19^ However, heterogeneity across the studies included in this meta‐analysis was high (*I^2^ *= 97.1%), primarily related to the sequence of performing unattended and attended BP measurements and the device used for automated office BP. Many studies included in this meta‐analysis did not randomize the patients but rather used alternating sequence of attended and unattended measurements in successive participants. Furthermore, comparison between unattended and attended resting automated office BP in some of these studies is derived from measurements within the same patient visit.^20^, ^21^, ^22^ In other words, the discordant measurements do not reflect standard clinical practice since the resting patients were subjected to BP measurements over more than 20 min taking into account two (attended and unattended) resting protocols each lasting 5 min and each followed by about 5 or more minutes of actual BP measurements. Hence, not surprisingly, large and potentially clinically relevant differences between attended and unattended (128 ± 15 vs. 134 ± 19 mm Hg, respectively) resting automated office BP readings did not translate into difference in correlation with target organ damage in the form of left ventricular hypertrophy and intimal media thickness.^22^ Our study arguably provides more robust data as the order of unattended versus partially attended automated office BP measurements was by randomization and at each clinical visit only one set of resting BP was taken, thus reflecting real life practice and eliminating BP lowering effect of prolonged resting.^23^ Furthermore, the design of our study allows us to compare partially attended measurements by two different devices, in particular the Omron HEM 907 and the BpTRU, between each other as well as to unattended measurements by Omron HEM 907 showing no statistical and clinically relevant differences. That leads to the second clinically relevant point that there is no absolute need to replace semi‐automated oscillometric BP devices (which do not allow for unattended BP measurements) with fully automated ones unless such replacement is required for other reasons. Furthermore, unattended resting, which requires a room for about 10 min and is unavailable for other purposes, may impose a logistical issue for many practitioners, and based on results of this study, for no obvious clinically relevant benefit.

Our second intention was to prove that resting office BP readings obtained by automated oscillometric devices are in agreement with daytime systolic BP readings obtained from 24‐h ABPM. Our findings, however, indicate the opposite. Resting office BP readings, unattended or partially attended, as obtained by automated or semi‐automated oscillometric devices, respectively, significantly underestimate overall daytime systolic BP load as assessed from 24‐h ABPM. Our results are in agreement with report by some, but not all groups.^24^, ^25^, ^26^, ^27^, ^28^, ^29^, ^30^ There is clinical heterogeneity between studies comparing these two methods including the time gap between the two tests of up to 1 month, time between actual BP readings during AOBP (1 vs. 2 min), number of AOBP readings (ranging from 3 to 5), populations studied (treated vs. untreated; men vs. women; age, etc.).^29^ None of these appeared to be materially different as reported by Roerecke and coworkers in meta‐analysis of 31 prospective clinical trials comparing unattended AOBP with daytime average BP by 24‐h ABPM. Roerecke, however, reported a significant difference [5.4 mm Hg (95% CI 1.7–9.1), *p* < .001, I^2^ 96%] between mean daytime systolic BP by 24‐h ABPM and automated office systolic BP in patients with controlled (SBP < 130 mm Hg) hypertension but no statistically and clinically relevant difference [0.3; 95% CI ‐1.1 to 1.7 mm Hg) between the two measurements among patients with “uncontrolled” hypertension (systolic BP ≥130 mm Hg). The results of the SPRINT ambulatory BP study are conceptually in agreement with the report by Roerecke and coworkers In the subset of 897 SPRINT participants who underwent 24‐h ABPM within 3 weeks of the 27‐month study visit, the systolic AOBP was 119 ± 12 mm Hg and daytime average systolic BP 126 ± 12 mm Hg in the intensive treatment group as compared to systolic AOBP of 135 ± 13 mm Hg and daytime average systolic BP 138 ± 12 mm Hg in the control group.^31^ In light of these findings, our data are derived from the cohort of patients with systolic AOBP in the range of 125–130 mm Hg (using different device and attended vs. unattended method of BP measurement), hence difference between systolic AOBP and mean daytime of about 10 mm Hg is in agreement with the reports included in the above meta‐analysis and results from the SPRINT ambulatory BP study. It is important to acknowledge that analyses showing difference between resting AOBP and daytime BP by 24‐h ABPM are derived from study level (and not from patient level) data and were not prespecified outcomes.

Our study cannot address the issue of whether this difference (around 10 mm Hg) in systolic BP readings obtained by AOBP and by 24‐h ABPM is clinically relevant. Observational studies do report that a greater difference between AOBP and daytime ABPM is associated with higher atherosclerotic cardiovascular disease risk score and a history of asymptomatic cardiovascular disease.^32^ There are, however, no prospective randomized controlled trials on cardiovascular outcomes in hypertensive patients managed purely using an ABPM‐based strategy. AOBP if properly applied largely eliminates white coat effect, but it does not eliminate masked hypertension nor can it adequately assess overall systolic BP load and variability during daytime activities and rest. As the thresholds for diagnosis and BP treatment targets are based on research quality office BP readings, we do not perceive comparison or equivalence of resting AOBP and daytime BP by 24‐h ABPM as necessary, but rather we suggest that AOBP should be considered complimentary to the 24‐h ABPM. If the difference between AOBP and daytime BP from 24‐h ABPM is higher in those with treated and controlled hypertension (as also seen in SPRINT), it makes the importance of a 24‐h ABPM in this group of patients particularly compelling.

The results of our study do show that the casual systolic office BP readings are significantly higher as compared to AOBP. The difference is similar whether compared to attended and unattended AOBP. Our data are in agreement with reports by others and reflected in consensus by national professional organizations that casual office BP should not be used for the diagnosis or management of HTN.

Diastolic BP readings varied significantly between two devices used in this trial, BpTRU and Omron HEM907XL. It appears that these differences are real, and in our opinion they reflect different algorithm for diastolic BP calculation from the oscillatory envelope which are specific to each manufacturer. These differences, while significant statistically, do not appear to be clinically relevant.

The major strength of our study is in its design as it in comparison to other studies resembles the most daily clinical practice applying only one set of AOBP methodology (attended or unattended) at given time, eliminated “order bias” as the order of attended versus unattended AOBP was by randomization, and eliminated the time gap between AOBP and 24‐h ABPM. The use of AOBP from two consecutive days largely eliminates the effect of first exposure during the study.

Our study has several limitations. Firstly, at the time this study was conceived and executed we were not aware of differences between systolic AOBP and daytime systolic BP from 24‐h ABPM described by Roerecke and coworkers and observed in the SPRINT ambulatory BP substudy.^29^, ^31^ Hence our data are relevant primarily to patients with systolic AOBP < 130 mm Hg. Secondly, due to protocol violations, the numbers in each group were not exactly 30 as planned. Despite this, the relative numbers in each group were robust enough and the differences in numbers between the groups small with regards to analysis of the primary outcome. Third, when interpreting this data, it is crucial to understand that even casual office BP in this study is of research quality with regards to execution and may not be generalizable to a casually performed BP in a busy primary care practice. Fourth, our data indicating no effect on presence of medical personnel during resting on AOBP should be interpreted with caution as “presence” does not equal presence of noise from moving or working and changes to lighting conditions etc.

Casual office BP readings are significantly higher compared to office BP readings after 5‐min rest. As treatment thresholds and BP targets are based on “SPRINT‐like” resting office BP, it is important to acknowledge that unattended resting may impose logistical issues for practitioners with no clinically meaningful impact on resting office BP. Casual and resting office BP readings by AOBP naturally cannot capture complexity of BP behavior and overall daytime and nocturnal BP load provided by 24‐h ABPM and as such, they should not be the sole diagnostic tool for diagnosis and management of hypertension.

## CONFLICTS OF INTEREST

None declared.

## AUTHOR CONTRIBUTIONS

Study conception and design: Swapnil Hiremath, Tim Ramsay, and Marcel Ruzicka. Analysis: Tim Ramsay and Swapnil Hiremath. Initial draft: Marcel Ruzicka and Swapnil Hiremath. Drafting the work or revising it critically for important intellectual content: Marcel Ruzicka, Tim Ramsay, and Swapnil Hiremath. Final approval of the version to be published: Marcel Ruzicka, Tim Ramsay, and Swapnil Hiremath

## Supporting information

Supporting Information
Supplementary Data
Supplementary Figure 1: Flow of the study
Supplemental Figure 2 : Bland Altman limits of agreement for pooled automated oscillometric systolic blood pressure compared to daytime ambulatory systolic blood pressure
