# Supplementary material for: Blood pressure measurement: Should technique define targets?
Source: J Clin Hypertens (Greenwich). 2021 Jul 16;23(8):1538–46. doi: 10.1111/jch.14324 (PMC8678755; doi:10.1111/jch.14324)
Supplement: Supplementary file 1 — Supporting Information Supplementary Data Supplementary Figure 1: Flow of the study Supplemental Figure 2 : Bland Altman limits of agreement for pooled automated oscillometric systolic blood pressure compared to daytime ambulatory systolic blood pressure [file JCH-23-1538-s001.docx]

**Supplemental Figure 1: Flow of the study**

**
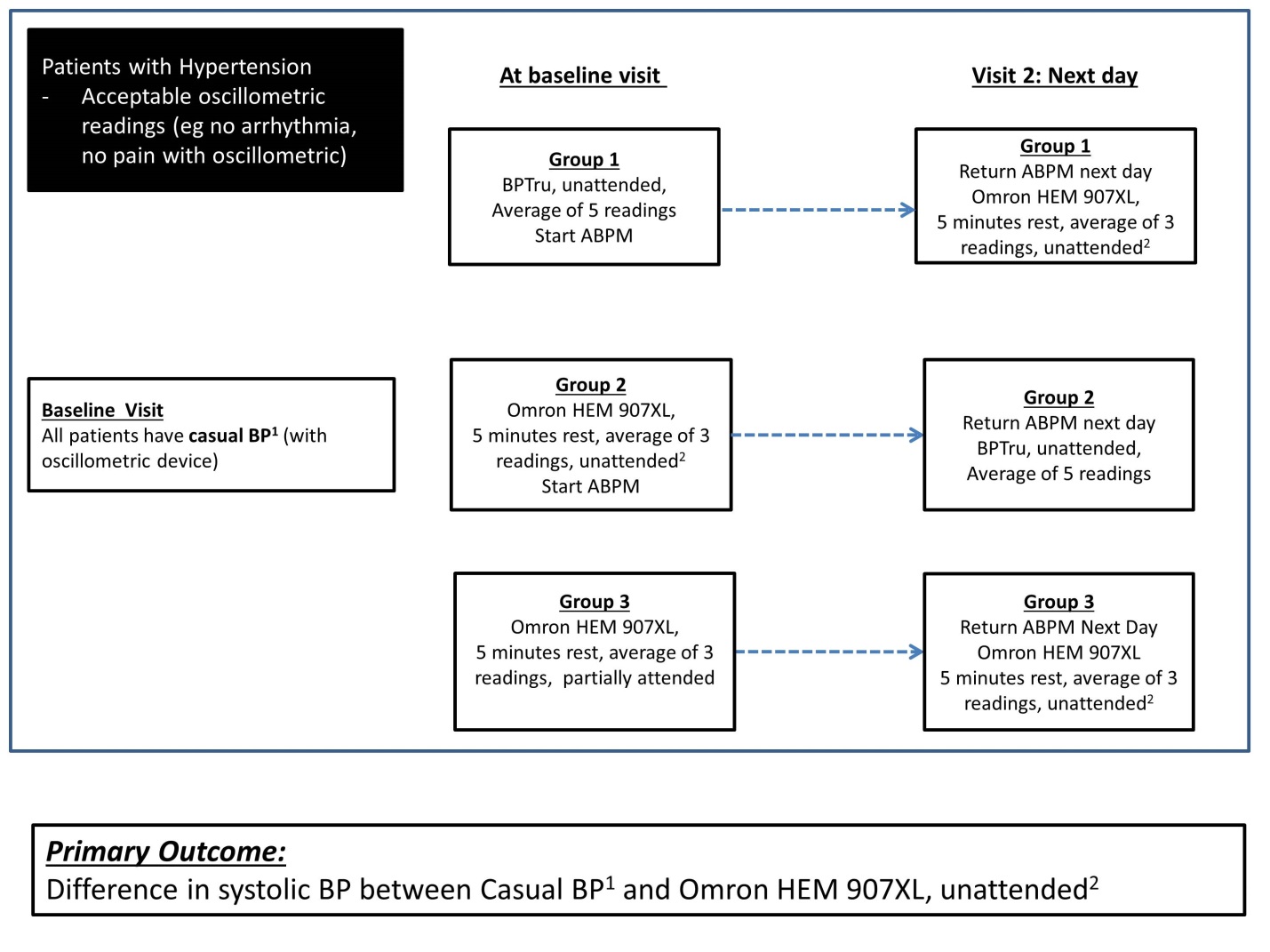
**

**Supplemental Figure 2: Bland Altman limits of agreement for pooled automated oscillometric systolic blood pressure compared to daytime ambulatory systolic blood pressure**

**
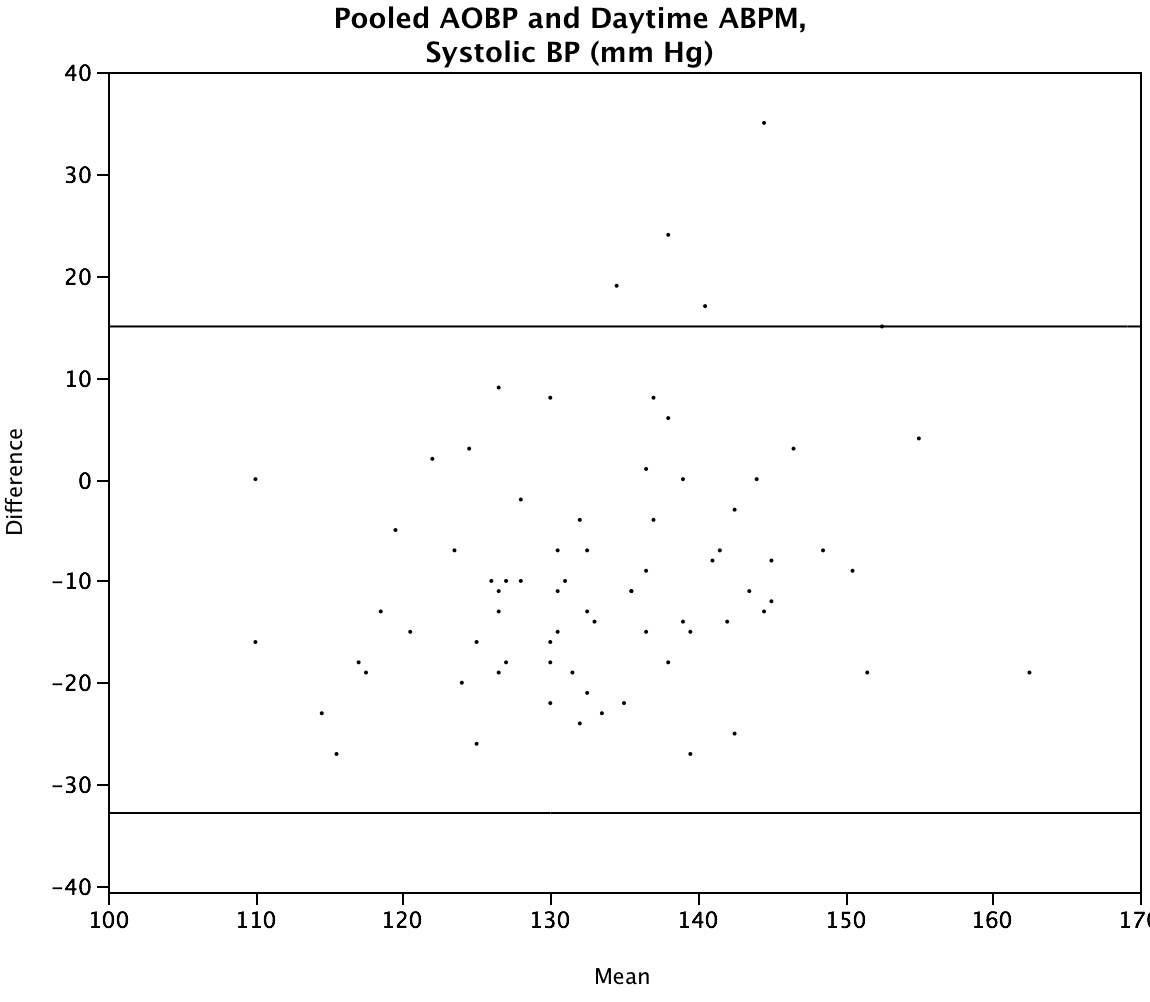
**
